# A Systematic Meta-Analysis of Global *Sarcocystis* Infection in Sheep and Goats

**DOI:** 10.3390/pathogens12070902

**Published:** 2023-07-02

**Authors:** Ying Feng, Ruiying Guo, Xiaoyu Sang, Xiaohan Zhang, Meiqi Li, Xiang Li, Na Yang, Tiantian Jiang

**Affiliations:** 1 Key Laboratory of Livestock Infectious Diseases in Northeast China, Ministry of Education, Key Laboratory of Zoonosis, College of Animal Science and Veterinary Medicine, Shenyang Agricultural University, Dongling Road 120, Shenyang 110866, China; myfengying@syau.edu.cn (Y.F.); fruiming@stu.syau.edu.cn (R.G.); xysang2016@syau.edu.cn (X.S.); zhangxiaohan7@stu.syau.edu.cn (X.Z.); limeiqi@stu.syau.edu.cn (M.L.); lixiang1998@stu.syau.edu.cn (X.L.); 2 Key Laboratory of Ruminant Infectious Disease Prevention and Control (East), Ministry of Agriculture and Rural Affairs, Shenyang 110866, China; 3 Department of Pediatrics, School of Medicine, University of California, La Jolla, San Diego, CA 92093, USA

**Keywords:** Sarcocystosis, *Sarcocystis* spp., infection rate, goat, sheep

## Abstract

Sarcocystosis is an intracellular parasitic disease caused by *Sarcocystis* spp. that has a worldwide prevalence. Symptoms of the disease include diarrhea and muscle pain. The disease poses a threat to the health of animals. The aim of this review is to investigate the global prevalence of *Sarcocystis* infection in sheep and goats during 2013–2022. We searched five databases: Web of Science, Science Direct, PubMed, Scopus, and Google Scholar. A total of 36 articles containing 44 datasets met the criteria and were included in the study. The total infection rates of *Sarcocystis* in sheep and goats were 66.3% (95% CI, 51.79–79.38%) and 52.1% (95% CI, 29.45–74.23%), respectively. It was found that *Sarcocystis* species tend to have a host species preference. Coinfection of *S. tenella* and *S. arieticanis* often occurred in sheep, and goats were frequently infected with *S. capracanis*. Age and sex were identified as risk factors for *Sarcocystis* infection in sheep and goats. The infection rates of female and male animals were significantly different, with females having a higher infection rate. Age-adjusted analysis showed that infection rates in animals older than one year were higher than in animals younger than one year. This study unveiled the global distribution of *Sarcocystis* and sheds light on its transmission in sheep and goats.

## 1. Introduction

Sarcocystosis is an intracellular parasitic disease with a worldwide distribution. *Sarcocystis*, the causative agent of the disease, is a two-host protozoan that can infect virtually all warm-blooded animals. There are more than 200 species of *Sarcocystis*, but only 26 species have known life cycles [1]. The definitive hosts of *Sarcocystis* are mostly predatory carnivores, while the intermediate hosts are mostly herbivores and omnivores. The intermediate hosts accidentally consume water or food contaminated with oocysts or sporocysts containing sporozoites. Upon digestion by gastric acid, the sporocysts containing sporozoites egress, migrate to the blood circulation, and travel to the muscles to form cysts. When the definitive hosts eat the intermediate hosts that harbor tissue cysts, the liberated bradyzoites migrate to the lamina propria of the small intestine and develop into male and female gametes. Male and female gametes fuse to develop into oocysts that are excreted in feces [2].

A variety of methods are now used to detect *Sarcocystis*, including visual inspection, microscopic examination of muscle squash, pepsin digestion, indirect immunofluorescence, histology, and polymerase chain reaction (PCR), including PCR, multiplex PCR, PCR-RFLP, and nested PCR [3,4,5,6,7,8]. The four genes that are commonly used for species identification are 18s rRNA, 28s rRNA, mitochondrial COX1 (cytochrome c oxidase subunit I), and ribosomal transcriptional space 1 (ITS-1) [9]. Among these, 18s rRNA and 28s rRNA are highly conserved and are often used for intraspecific identification. Mitochondrial COX1 and ITS-1 have strong specificity and are often used for interspecies identification [10,11]. Since there are many species of *Sarcocystis* and the structures of some species are difficult to distinguish, more accurate detection results require a combination of two or more methods. PCR in combination with cyst wall microscopy have commonly been used for species identification [9,12].

*Sarcocystis tenella*, *Sarcocystis arieticanis*, *Sarcocystis gigantea*, and *Sarcocystis medusiformis* are known to frequently infect sheep. The former two are pathogenic, transmitted by canids, and form small tissue cysts, while the latter two are nonpathogenic, transmitted by felids, and form large cysts that are visible to the naked eye [2,9]. Symptoms of *S. tenella* infection in sheep include anorexia, weight loss, fever, anemia, hair loss, abortion, premature birth, neurologic signs, myositis, and death [2]. *Sarcocystis capracanis*, *Sarcocystis hircicanis*, and *Sarcocystis moulei* are the species that frequently infect goats. The definitive hosts of the former two are canids, and those of the latter are felids. 

The economic importance of *Sarcocystis* in sheep and goats is underrated, as abortion and weight loss are often undiagnosed. In addition to health-related problems, *Sarcocystis* can result in low wool production [13]. To understand the global prevalence of *Sarcocystis* in sheep and goats, we conducted a meta-analysis, taking advantage of literature published in the last 10 years. We explored factors that influence *Sarcocystis* infection in goats and sheep, including age and sex. This review strives to elucidate the worldwide distribution and risk factors for *Sarcocystis* infection in goats and sheep, thus shedding light on the significant impact and transmission of this neglected parasitic disease.

## 2. Materials and Methods

### 2.1. Article Search

**Figure 1:**
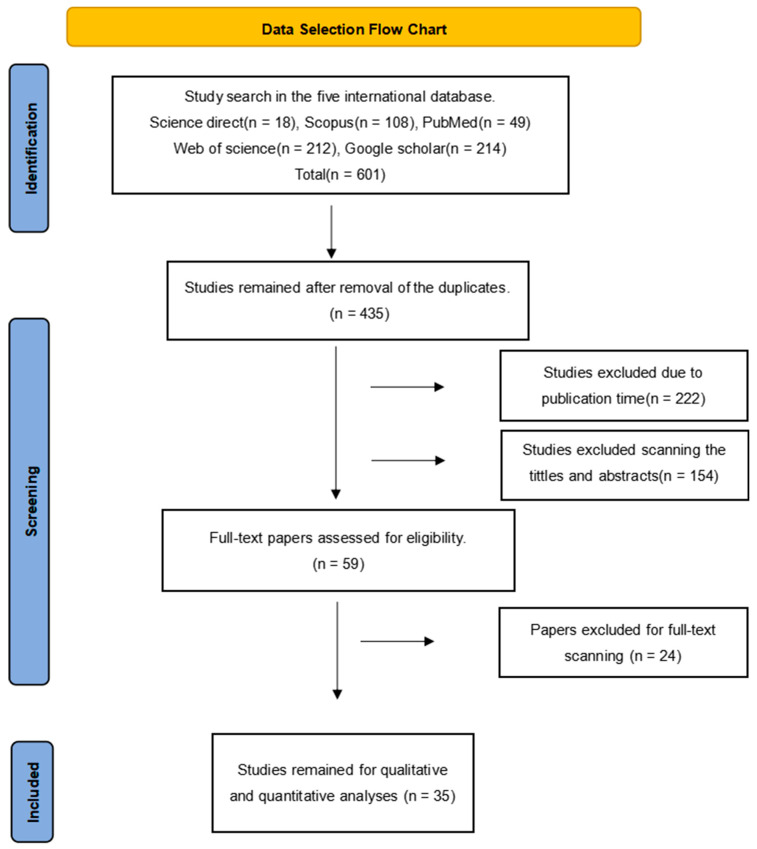
Data Selection Flow Chart.

The research was conducted strictly following PRISMA checklist (http://prisma-statement.org/PRISMAStatement/Checklist). A PRISMA flow diagram was created (Figure 1). In the five international databases (Web of science, Science di-rect, Pubmed, Scopus, and Google scholar), the following entries were used for article search, “sarcocystis spp.”, “sarcocystosis”, “sarcocysts”, “prevalence”, “epidemiology”, “frequency”, “occurrence”, “sheep”, “ovis ammon aries”, “ovis aries”, “ovis gmelini musimon”, “ovis aries musimon”, “goat”, “goats”, “capra”, “capras”. Retrieval was performed using “OR” and “AND” as Boolean operators.

### 2.2. Inclusion and Exclusion Criteria

Studies were included if they were sheep- or goat-related, descriptive studies or analytic cross-sectional studies, published between 2013 and 2022, written in English, containing data obtained through microscopy, serology, and/or molecular tools, and containing sample size and number of positive animals. The studies were excluded if they were case reports, letters, or reviews. Studies that evaluated infections in humans or animals other than sheep and goats and those that involved experimental infections were excluded. Surveys lacking total sample size, prevalence, or full texts were all excluded. 

### 2.3. Data Screening

After excluding duplicate articles and articles that had not been published in the last 10 years, the remaining articles were preliminarily scanned for titles and abstracts. Relevant data were extracted using an Excel spreadsheet containing the following items: first author, sampling time, sample size, infection rate, animal age, animal sex, data acquisition method, sampling site, and identified *Sarcocystis* species.

### 2.4. Data Extraction Formula

(((((((sheep[Title/Abstract]) OR (Ovis aries[Title/Abstract])) OR (Ovis ammon aries[Title/Abstract])) OR (Ovis gmelini musimon[Title/Abstract])) OR (Ovis aries musimon[Title/Abstract])) OR ((goats[Title/Abstract]) OR (Capras[Title/Abstract]))) AND ((((Prevalence[Title/Abstract]) OR (Epidemiology[Title/Abstract])) OR (Frequency[Title/Abstract])) OR (Occurrence[Title/Abstract]))) AND (((*sarcocystis* spp.[Title/Abstract]) OR (sarcocystosis[Title/Abstract])) OR (sarcocysts[Title/Abstract])). 

### 2.5. Statistical Analysis

In this study, R software was used to calculate the infection rates in sheep and goats. The *I*^2^ value was used to determine whether the meta-analysis should use a fixed or random effects model. The heterogeneity was divided into three grades of low, medium, and high, based on *I*^2^ values of 25%, 50%, and 75%, respectively. A fixed effects model was used for low heterogeneity, and a random effects model was used for medium and high heterogeneity. The prevalence was analyzed according to sex and age.

## 3. Results

**Figure 2:**
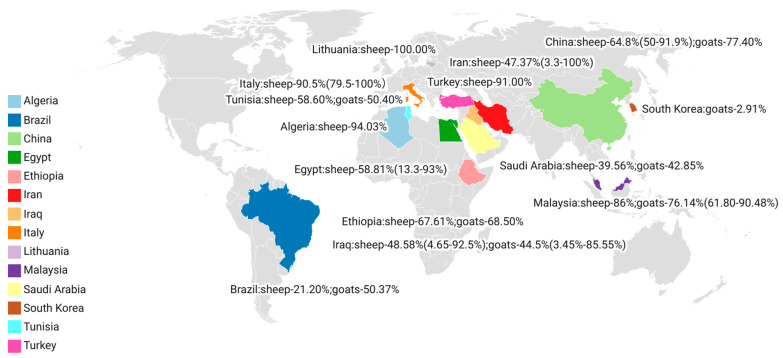
The global prevalence of *Sarcocystis* spp. infection in sheep and goats. Created with Datawrapper.

Five international databases were searched, which yielded a total of 601 articles, among which 18 articles were retrieved from Science Direct, 108 from Scopus, 49 from PubMed, 212 from Web of Science, and 214 from Google Scholar (Figure 1). A total of 166 duplicate articles were deleted, including 139 that were removed using EndNote X9 software, and 27 were deleted manually. Based on our time scope (2013–2022), 222 articles were excluded. After checking the abstracts and titles, 116 irrelevant articles were weeded out. The full texts of the remaining 59 articles were downloaded and carefully read. In the end, only 35 articles were included in this study. Forty sets of data were obtained, including 30 sets on sheep [5,9,13,14,15,16,17,18,19,20,21,22,23,24,25,26,27,28,29,30,31,32,33,34,35,36,37,38,39,40] and 10 on goats (Figure 2, Table 1 and Table 2) [16,17,36,37,38,41,42,43,44,45]. Meta analysis data can be downloaded from Supplementary Material.

**Figure 3:**
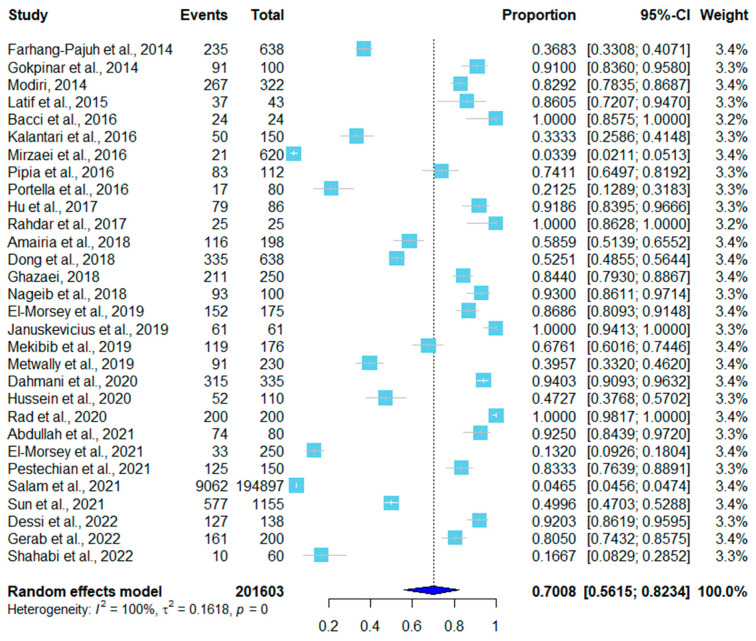
Forest plot of infection rates in sheep [5,9,13,14,15,16,17,18,19,20,21,22,23,24,25,26,27,28,29,30,31,32,33,34,35,36,37,38,39,40]. The proportion column calculated the ratio of the number of positive samples (events) to the total number of samples (total). The weight each study carried in the data analysis is listed in the last column.

**Figure 4:**
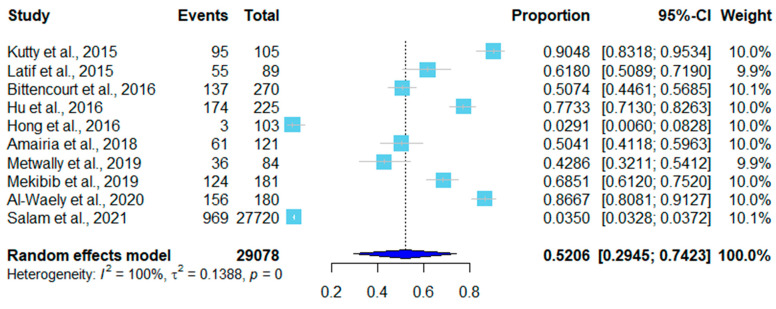
Forest plot of infection rates in goats [16,17,36,37,38,41,42,43,44,45]. The proportion column calculated the ratio of the nuber of positive samples (events) to the total number of samples (total). The weight each study carried in the data analysis is listed in the last column.

The infection rate of 201,603 sheep was 70.08% (95% CI, 0.5615–0.8234), and the infection rate of 29,078 goats was 52.1% (95% CI, 0.2945–0.7423), as shown in Figure 3 and Figure 4. Of the thirty studies in sheep, fifteen utilized the tissue squash method, nine used molecular techniques (PCR), two used histopathology, three used digestion, and one used Percoll gradient centrifugation. *Sarcocystis tenella* was identified in 18 studies, *S. arieticanis* infection in sheep was found in 13 studies, and 12 studies were mixed infections of *S. tenella* and *S. arieticanis* (Table 1).

Five of the ten studies on goats used tissue squash, four used PCR, and one used digestion. In six studies, the species was identified as *S. capracanis* (Table 2).

**Figure 5:**
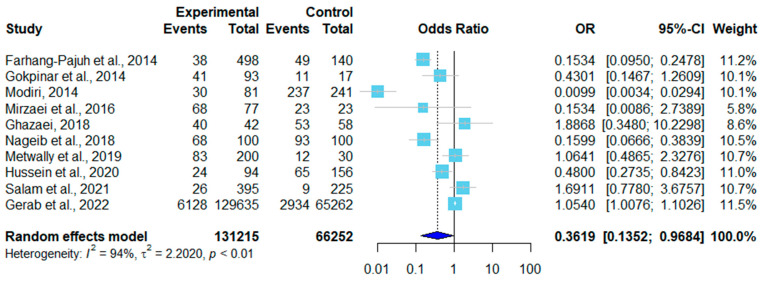
Forest plot of the infection rates in male and female sheep in different countries [20,23,25,26,30,31,34,36,37,40]. The experimental column compiled the number of positive samples over the total number of samples from male sheep. The control group compiled the number of positive samples over the total number of samples from female sheep. OR represents the odds ratio, which is the ratio of odds of infection in male and female sheep. OR = 1 is not statistically significant. The weight each study carried is shown in the last column.

**Figure 6:**
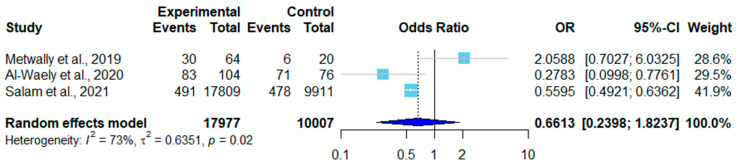
Forest plot of the infection rates in male and female goats in different countries [36,37,44]. The experimental column compiled the number of positive samples over the total number of samples from male goats. The control group compiled the number of positive samples over the total number of samples from female goats. OR (odds ratio) was the ratio of odds of infection in males and females. OR = 1 is not statistically significant. The weight each study carried is shown in the last column.

There were 12 sex-related datasets on sheep. Two were excluded due to a lack of male controls. In six of the remaining ten, females had higher infection rates than males (Table 3). A forest plot was drawn according to the odds ratio of infection in male and female sheep (Figure 5). In the random effects model, OR = 0.36, 95% CI (0.14–0.97) (Figure 4), *I*^2^ = 94%, and *p* < 0.01, indicating that there were significant differences in infection rates in sheep in different studies (Figure 5). Of the three sex-related datasets on goats, two showed a higher infection rate in male goats than in female goats (Table 4). As shown in Figure 6, a forest plot was drawn according to the odds ratio of infection in male and female goats. The random effects model showed that OR = 0.66, 95% CI (0.24–1.82), *I*^2^ = 73%, and *p* = 0.02, indicating that the infection rates of goats varied significantly among studies (Figure 6).

**Figure 7:**
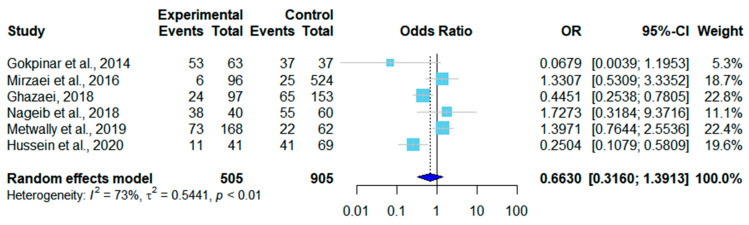
Forest plot of the age-adjusted infection rates in sheep from different studies [25,26,31,34,37,40]. The experimental group contained the number of positive samples and the total number of samples from sheep less than one year of age. In the control group, the number of positive samples and the total number of samples from sheep older than one year of age were included. OR (odds ratio) is the ratio of odds of infection in sheep less than and more than one year of age. OR = 1 is not statistically significant. The weight of each study is shown in the last column.

**Figure 8:**
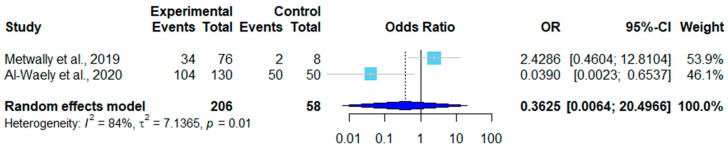
Forest plot of the age-adjusted infection rates in goats from different studies [37,44]. The experimental group contained the number of positive samples and the total number of samples from goats less than one year of age. The control group contained the number of positive samples and the total number of samples from goats older than one year of age. OR (odds ratio) is the ratio of odds of infection in goats less than and more than one year of age. OR = 1 is not statistically significant. The weight of each study is shown in the last column.

There were ten age-related datasets on sheep, among which six showed that the infection rate increased with age (Table 5). A forest plot was drawn according to the age-adjusted infection rates. In the random effects model, OR = 0.66, 95% CI (0.32–1.39), *I*^2^ = 73%, and *p* < 0.01, which means there was a significant difference in the rates of sheep infections among different studies (Figure 7). Goats had three age-related datasets, and the data were divided into two groups of ≤1-year-old and >1-year-old. Only two studies met the criteria. The infection rate of goats older than one year of age was higher than that of those younger than one year of age (Table 6). In the random effects model, OR = 0.66 and 95% CI (0.32–1.39). A forest plot showed *I*^2^ = 84% and *p* = 0.01, which means the age-adjusted infection rates varied significantly among studies (Figure 8).

## 4. Discussion

*Sarcocystis* is an intracellular protozoan with a global distribution. Infection is often asymptomatic in ruminants, but in severe cases, infection can lead to loss of appetite, anemia, abortion, premature birth, or difficulty breathing and death [13]. Sarcocystosis causes hair loss and a reduction in milk production in sheep. *Sarcocystis* costs the Spanish sheep industry an estimated EUR 20 million per year [46]. As a result, epidemiological studies of *Sarcocystis* in sheep and goats provide guidance to the farming industry and public health safety. Recently, two meta-analysis studies have been conducted on *Sarcocystis* infection in ruminants in China [47] and Iran [48]. The infection rates of *Sarcocystis* in ruminants in China and Iran were reported to be 65% and 74.4%, respectively. In another meta-analysis study, the global prevalence of *Sarcocystis* in cattle was reported to be 62.7% [49]. In our study, we focused on sheep and goats and compiled data obtained from 14 countries worldwide (Figure 2). 

This article compiled and analyzed data from 30 studies on *Sarcocystis* in sheep from 13 countries (three from Italy, one from Malaysia, one from Tunisia, nine from Iran, three from China, one from Brazil, five from Egypt, two from Iraq, and one from each of the following countries: Saudi Arabia, Ethiopia, Lithuania, Turkey, and Algeria) [5,9,13,14,15,16,17,18,19,20,21,22,23,24,25,26,27,28,29,30,31,32,33,34,35,36,37,38,39,40]. In addition, we included ten studies on *Sarcocystis* in goats from eight countries (two from Malaysia, two from Iraq, and one from each of the following countries: Tunisia, China, Brazil, South Korea, Saudi Arabia, and Ethiopia) [16,17,36,37,41,42,43,44,45]. The highest (100%) infection rate in sheep was found in Italy, Lithuania, and Iran [15,18,21,39], and the lowest (1.25%) infection rate was found in Iran [50]. Infection rates were associated with the sampling site and detection method. The highest (90.48%) infection rate in goats was reported in Malaysia [41], and the lowest (2.91%) infection rate was found in South Korea [45]. In Iran, one study that took samples from the diaphragm and esophagus of sheep found the infection rate to be 100% [18], while another study that took brain samples found the infection rate to be 1.25% [50]. In a study published by Kutty et al. in 2015, the same samples were subjected to different detection methods [41]. The infection rates were 52.38% (55/105), 43.8% (46/105), and 90.48% (95/105) using microscopic examination of muscle squash, H&E staining, and PCR, respectively [41].

Among the included articles, 13 reported the infection rate of *sarcocystosis* by sex (Table 3 and Table 4). Seven of the thirteen studies showed that females had higher infection rates than males, while the other six studies showed the opposite. Modiri et al. (2014) found that female sheep (98.34%, 237/241) had a higher infection rate than male sheep (37.03%, 30/81) [23]. Studies that showed a higher infection rate in male animals often had a larger sampling size of males than females. Salam et al. (2021) found that the infection rate difference between male goats (50.7%, 491/17809) and female goats (49.3%, 478/9911) [51] was not significant. However, with the same sampling size of female and male animals, Gerab et al. (2022) found that female sheep (93%, 93/100) had a higher infection rate than male sheep (68%, 68/100) [30]. Some scholars believed that the higher infection rate in females was because female animals often had compromised immunity during gestation and delivery, which increased their risk of infection [11].

Among the included studies, we found that in most articles, the infection rate was higher in animals older than one year (59.42%) as compared to those younger than one year (26.82%) (Table 5 and Table 6). This result is related to the increase in the probability of infection as animals age [52]. While a few studies have shown higher rates of infection in younger animals, this may be due to smaller sample sizes of adult animals. 

The current study is subject to a few limitations. First, we are unclear as to how the animals were raised. Animals raised in different environments will likely have different infection rates. Second, different detection methods offer varying sensitivity and specificity, which will affect the infection rate [44]. The Percoll gradient centrifugation method was less widely employed in the detection of *Sarcocystis*. Third, the obtained data resulted from one-time surveys and may not represent the infection rates at other times. Fourth, the infection rates of *Sarcocystis* in sheep and goats were not reported in many other countries around the world. 

The PCR detection method is fast, accurate, and suitable for routine laboratory practice. Compared with conventional PCR, nested PCR and PCR-RFLP have more steps and are more suitable for distinguishing species [14]. Microscopic examination of tissue pellets is among the most commonly used detection techniques, and complete cysts can be visualized under a microscope. However, it requires trained hands and is time-consuming and labor-intensive, which limits the number of samples that can be processed. The accuracy is in question because sometimes cysts are easy to miss [2]. 

The histopathological method is time-consuming and laborious, but the internal structure of the cyst can be clearly seen. This method is more suitable for the study of the entire cyst structure. In the digestion method, the cysts in the tissue are digested, and the release of bradyzoites from the cysts facilitates the detection of the parasite. However, because the cyst wall is dissolved, the species of *Sarcocystis* cannot be distinguished, and a complete cyst cannot be obtained [2]. The Percoll gradient centrifugation method has a high cost and is not suitable for epidemiological detection, but it is more suitable for purifying cysts. With this method, the morphology of the obtained cysts will not change [40].

This study is the first statistical analysis of the global prevalence of *Sarcocystis* in sheep and goats in the past 10 years (2013–2022). We investigated risk factors, including age and sex, for *Sarcocystis* infection in sheep and goats. The data analysis provided here enriches our understanding of the distribution and transmission of *Sarcocystis* in sheep and goats on a global scale. 

## 5. Conclusions

The total infection rates of *Sarcocystis* in sheep and goats worldwide were 70.08% (95% CI, 0.5615–0.8234) and 52.1% (95% CI, 0.2945–0.7423), respectively. Age and sex were identified as risk factors for *Sarcocystis* infection. Female sheep and goats had higher infection rates than male sheep and goats. In terms of age, animals older than one year of age had higher infection rates than those younger than one year of age. 

## Figures and Tables

**Table 1:** 32 datasets of infection in sheep from different countries.

No.	Country	Sampling Time	Positive/Total	Infection Rate	Detection Method *	Tissue	Species	Reference (year)
1	Iran	2011.2–2012.1	235/638	36.83%	PCR-RFLP	Esophagus Diaphragm Skeletal muscle	*S. gigantea* *S. medusiformis*	[20] (2014)
2	Iran	unknown	267/322	82.91%	Digestion method	Esophagus Diaphragm	unknown	[23] (2014)
3	Turkey	2011.2–2012.1	91/100	91.00%	Percoll gradient centrifugation	Skeletal muscle	*S. tenella* *S. arieticanis*	[40] (2014)
4	Malaysia	2013.2–2013.10	37/43	86.00%	Digestion method	Tongue Heart Diaphragm Esophagus Skeletal muscle	*S. ovicanis*	[16] (2015)
5	Italy	2013	83/112	79.50%	Nested PCR	Heart	*S. tenella* *S. arieticanis*	[14] (2016)
6	Italy	2014.3–2014.4	24/24	100%	Conventional PCR	Heart	*S. tenella*	[15] (2016)
7	Brazil	unknown	17/80	21.20%	Conventional PCR	Heart	*S. tenella* *S. arieticanis*	[29] (2016)
8	Iran	2013.9–2013.10	50/150	33.30%	Tissue squash method	Intra-abdominal Muscle Diaphragm	*S. gigantea* *S. moulei* *Sarcocystis* spp.	[24] (2016)
9	Iran	2013.4–2013.10	21/620	3.30%	Tissue squash method	Esophagus Diaphragm	unknown	[26] (2016)
10	Iran	unknown	25/25	100%	PCR-RFLP	Heart Tongue Diaphragm Skeletal muscle	*S. tenella* *S. capracanis*	[21] (2017)
11	China	2015.3–2015.11	79/86	91.90%	Tissue squash method	Esophagus Diaphragm Skeletal muscle Tongue Heart	*S. tenella* *S. arieticanis*	[9] (2017)
12	Iran	unknown	211/250	84.40%	Tissue squash method	Intervertebral muscle Esophagus Heart Tongue	*S. arieticamis* *S. tenella*	[25] (2018)
13	Tunisia	2016.5–2016.6	116/198	58.6%	Conventional PCR	Neck	*S. tenella*	[17] (2018)
14	China	2014.3–2017.10	335/638	52.51%	Tissue squash method	Heart	*S. tenella* *S. arieticanis*	[27] (2018)
15	Egypt	unknown	93/100	93.00%	Tissue squash method	Esophagus Diaphragm Tongue Skeletal muscle Heart	*S. arieticanis* *S. tenella*	[34] (2018)
16	Saudi Arabia	2016.3–2017.1	91/230	39.56%	Digestion method	Tongue Heart Skeletal muscle Diaphragm Esophagus	*S. tenella*	[37] (2019)
17	Ethiopia	2016.11–2017.5	119/176	67.61%	Tissue squash method	Heart Esophagus	unknown	[38] (2019)
18	Lithuania	2012–2014	61/61	100.00%	Tissue squash method	Esophagus Diaphragm Heart Neck Jaw Back	unknown	[39] (2019)
19	Egypt	2017.1–2018.2	152/175	86.80%	Tissue squash method	Esophagus Diaphragm Skeletal muscle Tongue Heart	*S. tenella* *S. arieticanis*	[32] (2019)
20	Algeria	unknown	315/335	94.03%	Histopathological method	Esophagus Diaphragm	*S. arieticanis* *S. tenella*	[5] (2020)
21	Iran	2018.10–2019.5	200/200	100%	Conventional PCR	Esophagus	*S. gigantea* *S. tenella* *S. arieticanis*	[18] (2020)
22	Egypt	2018.4–2019.3	52/110	47.27%	Tissue squash method	Heart	*S. arieticanis*	[31] (2020)
23	Iran	unknown	125/150	83.33%	Conventional PCR	Heart	*S. gigantea* *S. tenella*	[19] (2021)
24	China	2017.2–2018.11	577/1155	50.00%	Tissue squash method	Heart Diaphragm Esophageal	*S. gigantea*	[28] (2021)
25	Egypt	2018.1–2019.6	33/250	13.20%	Histopathologic examination	Esophagus Tongues Diaphragm Abdominal muscle Skeletal muscle Heart	*S. gigantea* *S. medusiformis*	[33] (2021)
26	Iraq	2020.5–2020.7	74/80	92.50%	Tissue squash method	Diaphragm Esophagus	*S. tenella* *S. arieticanis*	[35] (2021)
27	Iraq	2020.8–2021.1	9062/194,897	4.65%	Tissue squash method	Esophagus Diaphragm Heart	*S. gigantea* *S. medisiformis*	[36] (2021)
28	Italy	2019.4–2019.7	127/138	92.00%	Conventional PCR	Heart	*S. tenella*	[13] (2022)
29	Egypt	2020.7–2021.6	161/200	80.50%	Tissue squash method	Esophagus Tongue Heart Masseter Skeletal muscle	unknown	[30] (2022)
30	Iran	2019.3–2019.5	10/60	16.66%	Tissue squash method	Muscles ^#^	*S. moulei*	[22] (2022)

* All PCR methods targeted the 18s rRNA gene, ^#^ Specific muscle type unknown.

**Table 2:** 10 datasets of infection in goats in different countries.

No.	Country	Sampling Time	Positive/Total	Infection Rate	Detection Method *	Tissue	Species	Reference (year)
1	Malaysia	2014.1–2014.2	95/105	90.48%	Conventional PCR	Skeletal muscle	*S. capracanis*	[41] (2015)
2	Malaysia	2013.2–2013.10	55/89	61.80%	Tissue squash method	Tongue Heart Diaphragm Esophagus Skeletal muscle	*S. capracanis*	[16] (2015)
3	South Korea	2014.1–2014.8	3/103	2.91%	Conventional PCR	Heart	*S. tenella*	[45] (2016)
4	China	2014.7–2015.9	174/225	77.40%	Tissue squash method	Esophagus Diaphragm Skeletal muscle Tongue Heart	*S. capracanis * *S. hircicanis*	[42] (2016)
5	Brazil	2012.1–2013.6	137/270	50.37%	Conventional PCR	Tongue Esophagus Heart	*S. capracanis*	[43] (2016)
6	Tunisia	2016.5–2016.6	61/121	50.4%	Conventional PCR	Neck	*S. capracanis*	[17] (2018)
7	Saudi Arabia	2016.3–2017.1	36/84	42.85%	Tissue squash method	Tongue Heart Skeletal muscle Diaphragm Esophagus	*S. capracanis*	[37] (2019)
8	Ethiopia	2016.11–2017.5	124/181	68.50%	Tissue squash method	Heart Esophagus	unknown	[38] (2019)
9	Iraq	2019.10–2020.3	156/180	85.55%	Digestion method	Esophagus Diaphragm	unknown	[44] (2020)
10	Iraq	2020.8–2021.1	969/27,720	3.45%	Tissue squash method	Esophagus Diaphragm Heart	*S. moulei*	[36] (2021)

* All PCR methods targeted the 18s rRNA gene.

**Table 3:** Infection rates of male and female sheep in different countries.

Reference (year)	Country	Region	Total	Male	Infected	%	Female	Infected	%
[20] (2014)	Iran	Urmia	638	498	38	7.63	140	49	35
[23] (2014)	Iran	Boroujerd	322	81	30	37.03	241	237	98.34
[40] (2014)	Turkey	Kirikkale	100	77	68	88.3	23	23	100
[26] (2016)	Iran	Tabriz	620	395	26	6.58	225	9	4.0
[25] (2018)	Iran	Ardabil	250	94	24	26.96	156	65	73.03
[34] (2018)	Egypt	Assiut	100	42	40	95.2	58	53	91.4
[37] (2019)	Saudi Arabia	Riyadh	230	200	83	41.5	30	12	40
[31] (2020)	Egypt	Qena	110	93	41	44.08	17	11	64.70
[36] (2021)	Iraq	Sulaymaniyah	194,897	129,635	6128	67.6	65,262	2934	32.4
[30] (2022)	Egypt	Tanta	200	100	68	68	100	93	93

**Table 4:** Infection rates of male and female goats in different countries.

Reference (year)	Country	Region	Total	Male	Infected	%	Female	Infected	%
[37] (2019)	Saudi Arabia	Riyadh	84	64	30	46.87	20	6	30
[44] (2020)	Iraq	Wasit	180	104	83	79.80	76	71	93.42
[36] (2021)	Iraq	Sulaymaniyah	27,720	17,809	491	50.7	9911	478	49.3

**Table 5:** Age-adjusted infection rates in sheep in different countries.

Reference (year)	Country, Region	No. of Positive Samples	Positivity (%)	Age Groups (year)	No. of Samples	Age-Adjusted No. of Positive Samples	Age-Adjusted Positivity (%)
[40] (2014)	Turkey, Kirikkale	91	91	1 2 3 4	63 21 12 4	53 21 12 4	84.12 100 100 100
[26] (2016)	Iran, Tabriz	35	5.64	<1 1–3 >3	96 423 101	6 18 11	6.25 4.25 10.89
[25] (2018)	Iran, Ardabil	89	35.6	<1 >1	97 153	24 65	24.74 42.48
[34] (2018)	Egypt, Assiut	93	93	0.5–2 2–4 ≥4	40 41 19	38 38 17	95 92.7 89.5
[38] (2019)	Ethiopia, Hawassa	176	67.61	1.5–2.5	176	119	67.61
[37] (2019)	Saudi Arabia, Riyadh	95	41.30	<1 >1	168 62	73 22	43.45 35.48
[32] (2019)	Egypt, Gharbia	152	86.8	>2	175	152	86.8
[31] (2020)	Egypt, Qena	52	47.27	<1 >1	41 69	11 41	26.82 59.42
[28] (2021)	China, Qinghai	577	50	>1	1155	577	50
[30] (2022)	Egypt, Tanta	161	80.5	1–2 3–5	100 100	68 93	68 93

**Table 6:** Age-adjusted infection rates in goats from different studies.

Reference (year)	Country, Region	No. of Positive Samples	Positivity (%)	Age Groups (year)	No. of Samples	Age-Adjusted No. of Positive Samples	Age-Adjusted Positivity (%)
[37] (2019)	Saudi Arabia, Riyadh	36	42.85	<1 >1	76 8	34 2	44.73 25
[38] (2019)	Ethiopia, Hawassa	124	68.50	1.5–2.5	181	124	68.50
[44] (2020)	Iraq, Wasit	154	85.55	<1 1–2 >7	130 41 9	104 41 9	80 100 100

## Data Availability

The data presented in this study are available upon request from the corresponding author.

## Supplementary Materials

The following supporting information can be downloaded at: https://www.mdpi.com/article/10.3390/pathogens12070902/s1, File S1: meta analysis data.
